# Digital Interventions for Stress Among Frontline Health Care Workers: Results From a Pilot Feasibility Cohort Trial

**DOI:** 10.2196/42813

**Published:** 2024-01-09

**Authors:** Caroline W Espinola, Binh Nguyen, Andrei Torres, Walter Sim, Alice Rueda, Lindsay Beavers, Douglas M Campbell, Hyejung Jung, Wendy Lou, Bill Kapralos, Elizabeth Peter, Adam Dubrowski, Sridhar Krishnan, Venkat Bhat

**Affiliations:** 1 Department of Psychiatry University of Toronto Toronto, ON Canada; 2 Interventional Psychiatry Program, St. Michael’s Hospital Unity Health Toronto Toronto, ON Canada; 3 Department of Electrical, Computer, and Biomedical Engineering Toronto Metropolitan University Toronto, ON Canada; 4 maxSIMhealth Group Ontario Tech University Oshawa, ON Canada; 5 Allan Waters Family Simulation Program Unity Health Toronto Toronto, ON Canada; 6 Department of Physical Therapy University of Toronto Toronto, ON Canada; 7 Neonatal Intensive Care Unit, St. Michael’s Hospital Unity Health Toronto Toronto, ON Canada; 8 Li Ka Shing Knowledge Institute, St. Michael’s Hospital Unity Health Toronto Toronto, ON Canada; 9 Department of Pediatrics, Faculty of Medicine University of Toronto Toronto, ON Canada; 10 Dalla Lana School of Public Health University of Toronto Toronto, ON Canada; 11 Lawrence S. Bloomberg, Faculty of Nursing University of Toronto Toronto, ON Canada

**Keywords:** virtual reality, simulation, mobile app, stress, moral distress, moral injury, COVID-19, mobile phone

## Abstract

**Background:**

The COVID-19 pandemic has challenged the mental health of health care workers, increasing the rates of stress, moral distress (MD), and moral injury (MI). Virtual reality (VR) is a useful tool for studying MD and MI because it can effectively elicit psychophysiological responses, is customizable, and permits the controlled study of participants in real time.

**Objective:**

This study aims to investigate the feasibility of using an intervention comprising a VR scenario and an educational video to examine MD among health care workers during the COVID-19 pandemic and to use our mobile app for longitudinal monitoring of stress, MD, and MI after the intervention.

**Methods:**

We recruited 15 participants for a compound intervention consisting of a VR scenario followed by an educational video and a repetition of the VR scenario. The scenario portrayed a morally challenging situation related to a shortage of life-saving equipment. Physiological signals and scores of the Moral Injury Outcome Scale (MIOS) and Perceived Stress Scale (PSS) were collected. Participants underwent a debriefing session to provide their impressions of the intervention, and content analysis was performed on the sessions. Participants were also instructed to use a mobile app for 8 weeks after the intervention to monitor stress, MD, and mental health symptoms. We conducted Wilcoxon signed rank tests on the PSS and MIOS scores to investigate whether the VR scenario could induce stress and MD. We also evaluated user experience and the sense of presence after the intervention through semi–open-ended feedback and the Igroup Presence Questionnaire, respectively. Qualitative feedback was summarized and categorized to offer an experiential perspective.

**Results:**

All participants completed the intervention. Mean pre- and postintervention scores were respectively 10.4 (SD 9.9) and 13.5 (SD 9.1) for the MIOS and 17.3 (SD 7.5) and 19.1 (SD 8.1) for the PSS. Statistical analyses revealed no significant pre- to postintervention difference in the MIOS and PSS scores (*P*=.11 and *P*=.22, respectively), suggesting that the experiment did not acutely induce significant levels of stress or MD. However, content analysis revealed feelings of guilt, shame, and betrayal, which relate to the experience of MD. On the basis of the Igroup Presence Questionnaire results, the VR scenario achieved an above-average degree of overall presence, spatial presence, and involvement, and slightly below-average realism. Of the 15 participants, 8 (53%) did not answer symptom surveys on the mobile app.

**Conclusions:**

Our study demonstrated VR to be a feasible method to simulate morally challenging situations and elicit genuine responses associated with MD with high acceptability and tolerability. Future research could better define the efficacy of VR in examining stress, MD, and MI both acutely and in the longer term. An improved participant strategy for mobile data capture is needed for future studies.

**Trial Registration:**

ClinicalTrails.gov NCT05001542; https://clinicaltrials.gov/study/NCT05001542

**International Registered Report Identifier (IRRID):**

RR2-10.2196/32240

## Introduction

### Background

The COVID-19 pandemic has exerted unprecedented strain on health care workers (HCWs) globally [1]. Frontline HCWs have been forced to make difficult medical decisions that are contrary to their moral and professional principles and to work in conditions where they cannot meet standards of quality care [2,3], which has put them at a greater risk of experiencing moral distress (MD) than possibly ever before [4,5]. Distressing situations such as being forced to deal with a shortage of personal protective equipment and having to prioritize who will receive life-sustaining treatment have become common during the pandemic. For HCWs, experiencing such situations may cause significant emotional burden and induce the phenomenon of MD [6-8]. MD is defined as distress stemming from the inability to enact actions believed to be morally right owing to external constraints [8,9]. Moral injury (MI), an extreme form of MD, can occur when individuals witness or perpetrate actions that violate deeply held moral beliefs, resulting in severe emotional reactions with long-lasting consequences [7]. However, further investigation is needed to enable a more precise distinction between MD and MI [7].

The first description of MI was made in the military context by Shay [10] and was defined as a betrayal of moral character, usually as a result of the actions of a person in a position of authority [10], leading to feelings of powerlessness, helplessness, and loss of faith in humanity [7,10]. Shay [11] argues that MI occurs when the following conditions are met: (1) there has been a betrayal of what is considered right (2) by someone holding legitimate authority and (3) in high-stakes situations. Litz et al [12] expanded the concept of MI to include “the lasting psychological, biological, spiritual, behavioral, and social impact of perpetrating, failing to prevent, or bearing witness to acts that transgress deeply held moral beliefs and expectations.” As part of the definition, the authors also defined potentially morally injurious events (PMIEs) as the acts of perpetrating, failing to prevent harm, or bearing witness to acts that transgress deeply held moral beliefs [12]. Experiencing a PMIE is frequently associated with feelings of betrayal, guilt, shame, and self-blame [13]. Furthermore, PMIEs may not only cause acute MD but can also have long-term consequences because MD and MI may develop weeks or months after a PMIE [14].

MI was originally associated with, and frequently co-occurs with, posttraumatic stress disorder (PTSD) [13], which has been conceptualized as a fear-related disorder [15,16]. However, MI has not yet been defined in the Diagnostic and Statistical Manual of Mental Disorders, Fifth Edition [16], and a PMIE does not necessarily fulfill the Diagnostic and Statistical Manual of Mental Disorders, Fifth Edition criterion A for PTSD. The concept of MI was conceived to encompass the following criteria, among others: (1) reexperiencing self-referential moral emotions (eg, anger, guilt, and shame); (2) strong negative beliefs about the self, the world, and others; and (3) self-destructive behaviors that inflict severe distress or functional impairment [17,18]. In addition, emerging literature has defined MI as being mechanistically different from PTSD [13,15]. A positron emission tomography study in veterans with PTSD showed that regional blood glucose metabolism differed according to the nature of traumatic exposure as follows: the group with PTSD owing to danger-based traumas (ie, life-threatening events) showed higher metabolism in the amygdalae; by contrast, the group with PTSD secondary to non–danger-based traumas (eg, MI by self or others) had increased metabolism in the precuneus [19], a region that has been associated with the processing of self-referential feelings (eg, shame and guilt) [15]. Therefore, further research is needed to determine the ecological validity of MI as an independent diagnostic category [13]. In addition, there is a need to investigate specific interventions for MI because it has been found to not generally respond to evidence-based treatments for PTSD [12,17]; for example, moral resilience training, the development of emotional intelligence skills, and strategies for promoting moral repair have already been proposed as specific treatments for MI and are currently under investigation [5,17,20].

Although MI has been largely studied in military contexts [17], it is also applicable to HCWs, particularly in light of the COVID-19 pandemic. However, MI and PMIEs are poorly understood in this context. Čartolovni et al [7] argue that MI occurs in HCWs when they experience PMIEs involving high-stakes situations that are beyond their control. To investigate MI in the COVID-19 context, Rushton et al [5] conducted a survey with frontline HCWs and reported an overall prevalence rate of 32% for MI, with nurses being the most affected (38%). Fewer years of experience were positively associated with MI, whereas religious affiliation or spirituality and higher levels of moral resilience were associated with lower MI scores. In addition, the study showed a moderate correlation between MI and various ethically challenging situations, such as experiencing negative consequences at work after expressing safety concerns, working with limited resources, and carrying out decisions of others which threaten one’s own values [5].

Litam and Balkin [4] examined the relationship between MI and the professional quality of life in a convenience sample of HCWs during the COVID-19 pandemic. The authors reported that secondary traumatic stress was a strong predictor of MI in frontline HCWs, but the contribution of compassion, satisfaction, and burnout to MI scores was nonsignificant. Of note, nurses had significantly higher burnout scores than physicians. Zerach and Levi-Belz [21] conducted a survey to investigate the patterns of exposure to PMIEs in a sample of HCWs and social care workers during the COVID-19 pandemic. The prevalence rate of symptoms of MI was 40%, with betrayal events being the most frequent PMIEs with a prevalence rate of 62%. In general, exposure to PMIEs was positively related to perceived stress, depression, anxiety, and self-criticism, whereas it was negatively associated with self-compassion. Interestingly, the duration of care for patients with COVID-19 was not associated with MI [21].

To increase the ecological validity of MI as a diagnostic entity, the experiences of the MD-MI continuum should be examined using accurate methods [13]. To date, several measurement instruments have been developed to identify MI outside of military contexts, including the Moral Injury Symptom Scale–Healthcare Professionals version [22] and the Moral Injury Outcome Scale (MIOS) [18]. The MIOS is a self-rated scale, developed as an assessment tool to evaluate MI as a multidimensional outcome [18]. This scale comprises 10 binary (*yes* or *no*) questions and 15 five-point Likert scale questions about experiencing a PMIE and feelings associated with this event; higher scores indicate greater severity of MI symptoms. At the end, the MIOS has an additional 7-point Likert scale question that assesses the extent to which the experience of PMIEs has interfered in one’s self-care or caused functional impairment (from *not at all* to *extremely*). The MIOS is in the final stage of development by the MIOS Consortium [18].

Conducting interventional studies to investigate the impact of PMIEs on mental health in real-world settings is challenging owing to operational constraints. This is especially true in health care, where limitations imposed by patient privacy regulations may make traditional clinical trials in MI impractical. Another important aspect to consider is the ethical implications of submitting an already strained workforce to moral stressors in an uncontrolled real-world environment such as an intensive care unit (ICU). A promising strategy to address these limitations is the use of virtual reality (VR) scenarios. VR is a powerful technology for examining mental health and the MD-MI continuum because it offers several advantages over traditional observational research in naturalistic environments. First, VR allows researchers to observe, monitor, and potentially support participants in fully controlled environments in real time [23]; therefore, it is safer and provides more accurate measures of one’s reactions to ethically challenging situations compared with observational studies in naturalistic environments. Second, VR allows for the design of fully customizable scenarios [23], making it especially suitable to simulate real-world scenarios in health care that otherwise would be impractical to replicate. As traumatic events in both PTSD and MI are highly idiosyncratic, and treatment for PTSD requires exposure to individual cues, we assume that virtual environment customization should be a critical feature to provide personalized and effective interventions to treat MD and MI [24]. In addition, extensive evidence has demonstrated the effectiveness of VR-based interventions for PTSD [25-27]. Third, VR environments can effectively elicit real psychophysiological responses because individuals are immersed in virtual scenarios as if these were real events, with the advantage of enabling real-time data capture [23,24]. All these advantages make VR-based trials ideal to study the MD-MI phenomena in HCWs. However, no prior research has investigated the feasibility of VR interventions to examine MD and MI in the context of the COVID-19 pandemic.

### Objectives

The overarching goal of this study was to determine the feasibility of using a compound VR intervention to examine MD and MI among HCWs during the COVID-19 pandemic. To achieve this, we designed a VR scenario in which HCWs faced a morally challenging situation in a midpandemic hospital environment while being monitored for acute psychological and physiological measures of stress. As outlined in our protocol paper [28], our aims were to (1) evaluate the feasibility of using a VR scenario to simulate the experience of a COVID-19–related morally challenging event by using measures of tolerability, dropouts, and suitability of the virtual scenario; (2) assess the potential of our VR scenario to elicit mild stress and MD, as measured by quantitative self-report questionnaires as well as qualitative analyses of semistructured interviews; and (3) investigate the feasibility of our novel mobile app (*DiiG App*) for longitudinal monitoring of stress and MD in naturalistic settings in the 8 weeks after the intervention.

On the basis of the findings with PTSD [25-27], we hypothesized that VR scenarios would be a feasible method for assessing MD and MI. Given the ability of VR to generate genuine responses, we additionally hypothesized that our virtual scenario would significantly increase stress levels and elicit feelings and symptoms associated with MD and MI. Finally, we hypothesized that our mobile app would successfully capture symptoms associated with stress and MD in the 8-week follow-up.

To the best of our knowledge, this pilot study is the first to assess the feasibility of using a VR scenario to simulate the experience of a morally challenging event related to the COVID-19 pandemic by HCWs while assessing its acute perceptual, psychological, and physiological effects in real time.

## Methods

### Study Design

**Figure 1:**
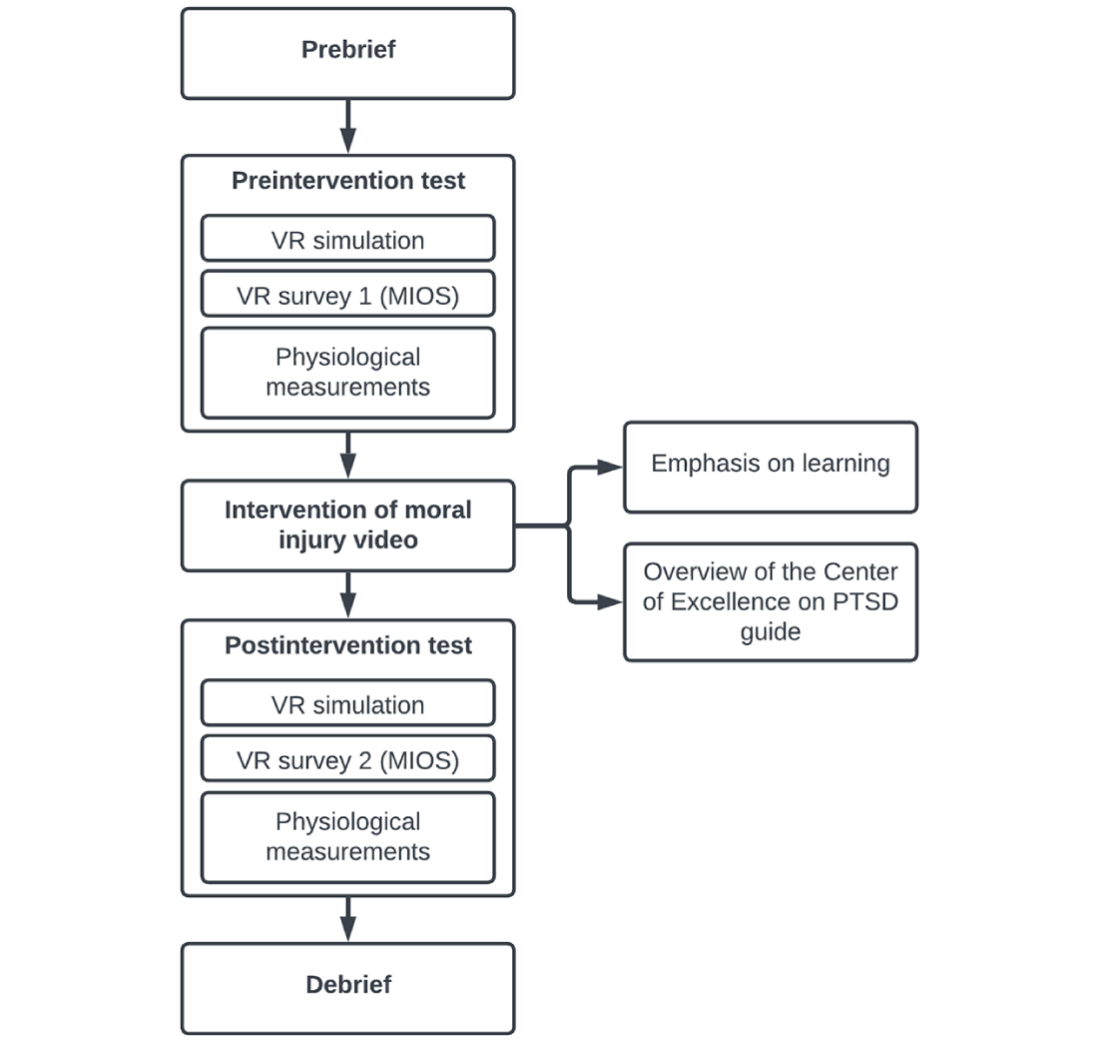
Flowchart of the virtual reality (VR) experiment. MIOS: Moral Injury Outcome Scale; PTSD: posttraumatic stress disorder.

In this single-cohort pilot study (ClinicalTrials.gov: NCT05001542), we adopted a multimethod approach in a pretest-posttest design to develop a compound intervention consisting of three successive parts: (1) a VR scenario to simulate a morally complex situation, (2) an educational video on MI and appropriate mitigation strategies, and (3) a repetition of the VR scenario. The intervention was followed by longitudinal data collection of mental health and MI surveys using our mobile app. The MI educational video was based on the Center of Excellence on PTSD guide [29] that summarized the causes and identifiers of MI and potential interventions to mitigate MD. The effectiveness of the VR-based educational intervention was assessed using the MIOS [18], the Perceived Stress Scale (PSS) [30], and the Igroup Presence Questionnaire (IPQ) [31]. The PSS is a self-reported measure of stress, whereas the IPQ evaluates the experience of presence during the VR scenario. As previously mentioned, the MIOS is a self-rated scale that was developed as an assessment tool to evaluate MI. For the purposes of this pilot study, we adopted a brief version of the MIOS (hereinafter referred to as *the MIOS*), which comprises 10 five-point Likert scale questions and 4 binary (*yes* or *no*) questions [32]. During the VR scenario, respiratory impedance, electrocardiography (ECG), galvanic skin response, and photoplethysmography were continuously collected. In addition to the original signals, we extracted the derivation of these signals, including ECG pulse rate, ECG RR interval, respiratory rate, and elevated respiratory rate. A visualization of the VR experimental flow can be seen in Figure 1. Further details on the intervention and data collection have been explained and outlined in the paper by Nguyen et al [28].

**Figure 2:**
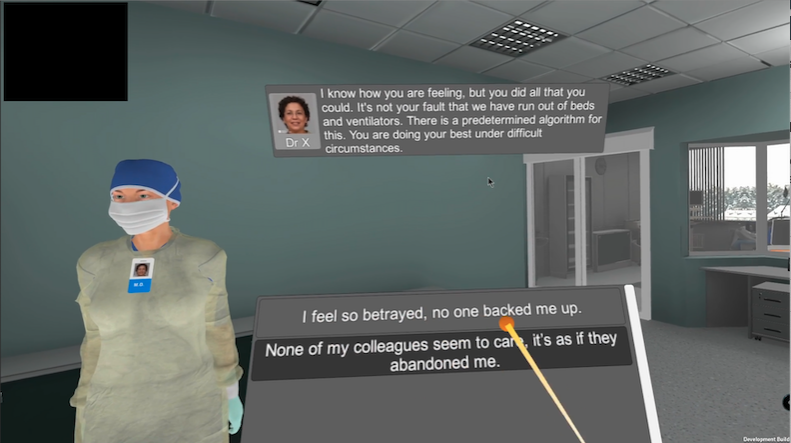
User interface displaying the dialogue and interaction panels.

The experimental session was divided into prebrief, preintervention test, intervention video, postintervention test, and debrief components (Figure 1). The preintervention test and postintervention test were conducted in VR, whereas the prebrief and debrief occurred outside the virtual environment. The MIOS was performed at 4 time points as follows: as a paper-based version for the prebrief and debrief and in the virtual scenario for the preintervention test and postintervention test. The PSS was performed twice, at prebrief and debrief. The MIOS and the PSS focus on symptoms of MD and stress, respectively, over the last month. However, when answering these scales, participants were told to rate symptoms at that exact moment. The goal of the prebrief was to explain how the physiological data would be collected and prepare the participant for the VR scenario; it consisted of an orientation to the virtual space and equipment, safety precautions, and the expected outcome of the study. During the preintervention test, participants were immersed in the VR scenario where they took on the role of a physician in an ICU during the COVID-19 pandemic. To experience the VR scenario, participants used a VR headset and 2 wireless controllers that tracked their head and hand movements, mapping it to an avatar. Semitranslucent panels were displayed as spatial elements in the VR scenario (Figure 2), providing information to the participant in the form of the dialogue panel (which displayed the current nonplayable character’s photograph, name, and the text version of the dialogue being spoken) and the interaction panel (which displayed a list of available choices and responses for the participant to choose from).

In the scenario, a shortage of life-saving equipment resulted in the decision to move a ventilator from 1 patient to another patient who had a greater chance of survival. After being informed of this, the participant’s avatar appeared in the next scene, where they had to communicate this decision to the first patient’s family and respond to the family’s reactions of frustration and anger. After completing the preintervention test and while still immersed in the VR scenario, participants watched a brief 2D educational video comprising key concepts of MD and MI and adaptive behaviors to cope with morally complex situations at the individual, team, and organizational levels. Participants then completed the postintervention test, which consisted in a repetition of the VR scenario played in the preintervention test. Finally, in the debrief, participants were asked open-ended questions to encourage them to describe their experiences in the virtual setting, followed by an exit survey [28].

After the experiment, participants were instructed to use our mobile app [33] to collect passive and active data for distress monitoring during the following 8 weeks. As MI may have a delayed onset, such data collection allows for longer-term monitoring of emotions associated with MD, offering insights into the distress experienced in real time.

### Participants

Participants were recruited and enrolled between May 2021 and August 2021 from the 3 affiliated hospitals at Unity Health Toronto. Participants were enrolled if they were an HCW currently providing health care at their respective hospital of employment, aged ≥18 years, and owned a mobile phone (an Android mobile phone with operating system version 6.0 or above or an iPhone with operating system version 11.0 or above).

### Statistical Analysis

As this was a pilot feasibility trial, we summarized dropout rates, easiness of use, tolerability, acceptability, and utility using counts and percentages. Continuous data were summarized using range, mean and SD, and median and IQR. To assess the effect of the VR scenario on symptoms of MI, we compared MIOS scores across the 4 time points using a Friedman test. In addition, follow-up MIOS scores were compared with the score at prebrief using Wilcoxon signed rank tests with Bonferroni correction (.05/3=.0167) to adjust for multiple comparisons. As PSS scores were collected only at 2 time points (ie, at prebrief and debrief), a Wilcoxon signed rank test was used to compare the difference in the PSS scores between these 2 time points. A *P* value of <.05 was considered significant unless otherwise specified. We performed statistical analysis using SAS 9.4 (SAS Institute Inc).

### Quantitative Analysis

#### Stress and MD Analysis

In this feasibility study, we piloted the application of the MIOS to assess MD both acutely and longitudinally. As mentioned in the *Study Design* section, MIOS was administered during the prebrief, preintervention test, postintervention test, and debrief. Participants were also prompted to complete MIOS on the mobile app in the 8 weeks after the intervention for a longitudinal assessment of MD and MI. All questionnaires used in the mobile app (eg, the MIOS and the PSS) are available in the appendices of the study by Nguyen et al [28].

#### IPQ Assessment

To objectively assess user experience within the VR scenario, we adopted the IPQ, which is a questionnaire for measuring the sense of presence experienced in a virtual environment [31]. Composed of 14 questions (answered on a 6-point Likert scale), the IPQ has a high reliability (Cronbach α=.87) and outputs four items (1 general item, not belonging to a subscale, and 3 subscales): (1) general presence (sense of being there), (2) spatial presence (the sense of being physically present in the virtual environment), (3) involvement (measuring the attention devoted to the virtual environment), and (4) experienced realism (measuring the subjective experience of realism in the virtual environment).

Hereinafter, the 4 outputs will be referred to as *IPQ components*. More information about the construction and structure of the scale and the IPQ’s reliability analysis is available on the Igroup project consortium website [34,35].

#### Mobile Data Analysis

After participating in the intervention, participants were instructed by our research staff to download and regularly use our mobile app to answer surveys in the 8-week follow-up. Participants received push notifications on the mobile app 3 times weekly to answer short versions of the scales related to depression (2-item Patient Health Questionnaire), anxiety (2-item Generalized Anxiety Disorder), stress (4-item PSS) MI (4-item MIOS), and loneliness (3-item University of California Los Angeles Loneliness Scale). With the exception of the 3-item University of California Los Angeles Loneliness Scale, participants were also asked to answer the full version of these scales once weekly. Short versions of the scales were used on weekdays to minimize participant burden. The mobile app also had the option of collecting passive data from built-in smartphone sensors (GPS and accelerometer) from participants who provided in-app consent to gather information on distance traveled and activity patterns. Details on the mobile data collection were previously overviewed in the study by Nguyen et al [28]. We used in-app automated survey reminders to promote app use.

### Qualitative Analysis

#### Content Analysis

**Figure 3:**
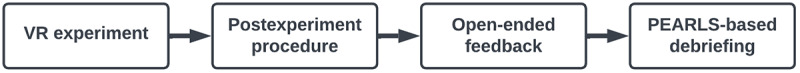
Flow diagram of the debriefing. PEARLS: Promoting Excellence and Reflective Learning in Simulation; VR: virtual reality.

We performed a content analysis on the data collected from the scenario debriefing conducted immediately after the compound intervention. Qualitative content analysis is a method to interpret meaning from text data and draw conclusions from words, themes, or concepts that occur in the text, in reference to their context, so that research questions can be answered [36]. We used inductive category development by becoming immersed in the data and allowing insights on categories to emerge from the data [37]. The scenario debriefing consisted of a semistructured interview that allowed participants to answer open-ended questions about their overall experience, followed by a semistructured debriefing methodology (the interview guide is included in Multimedia Appendix 1). The researchers (BN and AT) who collected the VR data were trained using the Promoting Excellence and Reflective Learning in Simulation (PEARLS) health care debriefing tool [38], a simulation debriefing framework to help learners assess their experience within a safe environment. A flow diagram of the debriefing can be seen in Figure 3. After completion of the intervention, we conducted a postexperiment procedure, which consisted of removing the VR headset from the participant but keeping the physiological sensors attached. In addition, we confirmed with the participant that they were able to continue with the debriefing.

During the open-ended feedback part of the debrief, we asked participants to speak freely about their experience with the experiment. We specifically asked the following questions:

1. “What suggestions or feedback would you give to improve the scenarios? Please comment on what can be improved, what can be more realistic, and any deviation from real-life applications.”
2. “Could you share something that you have learned about moral injury today? How might this apply to your clinical practice?”

The research questions we sought to answer with our content analysis from this feedback were as follows:

1. “How can the VR scenario be improved?”
2. “How accessible and relevant was our intervention?”

We subsequently conducted scenario-based debriefing using the PEARLS [38] methodology, which involved an exploration of the following predetermined topics: participant experience with the technology used, decision-making during the scenario, and emotions elicited during the scenario. The research questions we sought to answer with the content analysis from the scenario-based debriefing were as follows:

1. “What is the overall user experience of participants with the VR technology?”
2. “What were the determining factors for the decisions that participants made in the scenario?”
3. “How did the scenario make the participant feel?”

The PEARLS structure is a well-validated debriefing tool that is typically used to provide introspection on performance for a simulation participant [38]. It has been used extensively in the simulation literature, including a recent user qualitative study with patient-led simulations [39]. A PEARLS debrief integrates 4 main segments: setting the scene, eliciting reactions, description and analysis of the experience, and summary or reflections.

After the debrief, participants were asked to answer a debrief feasibility questionnaire with 3 five-point Likert questions answered on a scale ranging from 1 (*strongly disagree*) to 5 (*strongly agree*) about the relevance and utility of the psychoeducational content on MD for real-life situations as well as the ability of the VR scenario to elicit emotions (Multimedia Appendix 1).

#### User Experience

**Figure 4:**
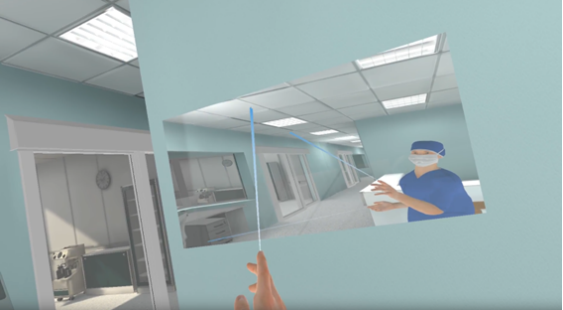
Snapshot of the virtual reality scenario showing the participant’s avatar reflected in a mirror. The blue beam indicates the cursor used to interact with the virtual environment.

To assess the user experience within the VR scenario, we evaluated the dropout rate, the feasibility questionnaire, and the qualitative responses provided during the debrief. During the VR scenario, participants had their head and hand movements tracked by the VR headset and controllers, and all movements were mapped into a virtual avatar (Figure 4). To help improve the sense of body ownership (ie, making the users recognize the virtual body as their own) [40], the preintervention test started with a tutorial that had the participants looking at a mirror and moving their head and hands to visualize that their virtual avatar actions reflected their own.

### Ethics Approval

Ethics approval was obtained from the research ethics board at St. Michael’s Hospital before starting any study activities (21-066).

## Results

### Participants

#### Participant Flow

**Figure 5:**
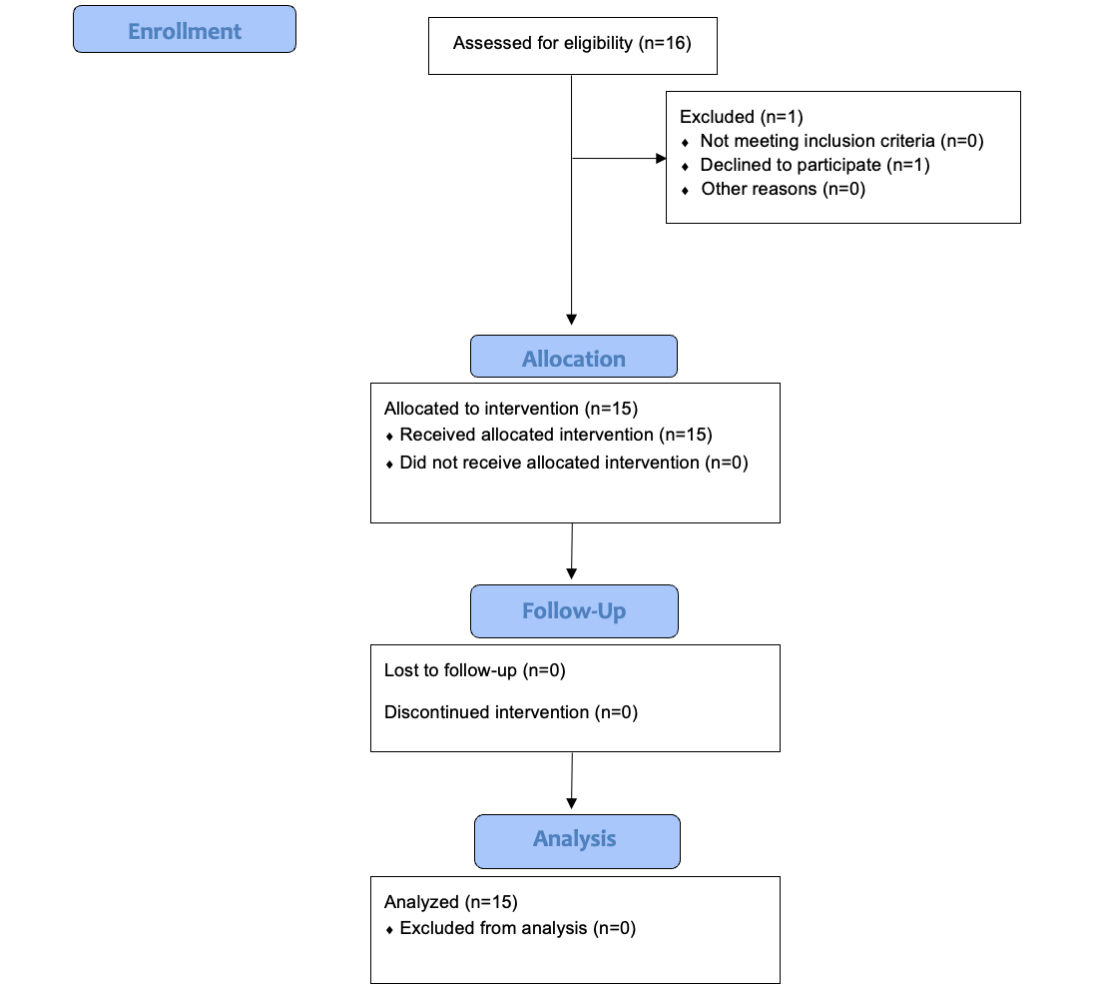
Flowchart of participant enrollment and attrition.

A total of 16 participants were assessed for eligibility; 1 (6%) declined to participate, and therefore 15 (94%) participants were allocated to the intervention. All 15 participants received the intervention. No participants were lost to follow-up, and data from all 15 participants were analyzed. Information on participant flow is presented in Figure 5.

#### Baseline Data

Our sample consisted of 15 HCWs (female participants: n=11, 73%; male participants: n=4, 27%). The participants had a mean age of 32.7 (SD 9.5) years; the male participants had a mean age of 34.3 (SD 4.9) years, whereas the female participants had a mean age of 32.2 (SD 10.9) years. Among the 15 participants, the most common occupations were nursing (n=7, 47%) and medicine (n=3, 20%); other professions included mental health research staff (n=2, 13%), physician assistant (n=1, 7%), educator (n=1, 7%), and graduate student (n=1, 7%). At the time of the experiment, none of the 15 participants had a prior or current COVID-19 infection; however, 4 (27%) had a prior family history of COVID-19 infection. The VR experiments were conducted between May 2021 and August 2021.

#### Data Analyzed

For 15 participants, MIOS, PSS, IPQ, and mobile data were analyzed. The data of 14 participants were analyzed for the content analysis.

### Quantitative Analysis

#### Stress and MD Analysis

The average MIOS scores for the prebrief, preintervention test, postintervention test, and debrief were 10.4 (SD 9.9), 12.9 (SD 6.9), 12.6 (SD 7.1), and 13.5 (SD 9.1), respectively, with a difference between the debrief and prebrief (between after the intervention and before the intervention) of 3.1 (SD 6.8; Table 1). There was no statistical difference in the MIOS scores at the 5% level when comparing all 4 scores using the Friedman test (Q=4.61; *P*=.20). Using Bonferroni correction (.05/3=.0167), the results showed no significant difference between the prebrief scores and any follow-up score: preintervention test (*P*=.30), postintervention test (*P*=.32), and debrief (*P*=.11). The MIOS is a new scale that is still under development by the MIOS Consortium and has not yet been established for the assessment of MI [18,41].

**Table 1:** Wilcoxon signed rank test comparing Moral Injury Outcome Scale follow-up scores at preintervention test, postintervention test, and debrief with the prebrief score (n=15)^a^.

	Values, mean (SD)	Values, median (IQR; range)	*P* value
Prebrief score	10.4 (9.9)	12 (0 to 17; 0 to 28)	N/A^b^
Preintervention test score	12.9 (6.9)	13 (6 to 17; 3 to 27)	.30^c^
Postintervention test score	12.6 (7.1)	13 (8 to 17; 1 to 28)	.32^c^
Debrief score	13.5 (9.1)	14 (5 to 18; 0 to 32)	.11^c^
Difference (debrief − prebrief)	3.1 (6.8)	1 (−1 to 7; −8 to 18)	.11^c^

^a^There was no statistical difference in the Moral Injury Outcome Scale scores at the 5% level when comparing all 4 scores using the Friedman test (Q=4.61; *P*=.20).

^b^N/A: not applicable.

^c^Follow-up scores were compared with the preintervention test score using the Wilcoxon signed rank test; Bonferroni correction was used (.05/3=.0167), that is, significance at 1.67% was applied.

PSS scores were only collected at 2 time points: at prebrief and debrief. The average PSS scores during the prebrief and the debrief were 17.3 (SD 7.5) and 19.1 (SD 8.1), respectively, with a postintervention test–preintervention test difference of 1.8 (SD 6.0; Table 2). Similar to the MIOS scores, the prebrief and debrief PSS scores were not statistically different (*P*=.22). Tables 1 and 2 summarize the analysis for the MIOS and PSS scores.

**Table 2:** Wilcoxon signed rank test of the Perceived Stress Scale prebrief and debrief scores (n=15).

	Values, mean (SD)	Values, median (IQR; range)	*P* value
Prebrief score	17.3 (7.5)	15 (12 to 22; 4 to 33)	N/A^a^
Debrief score	19.1 (8.1)	19 (14 to 26; 4 to 33)	N/A
Difference (debrief − prebrief)	1.8 (6.0)	1 (−1 to 7; −11 to 11)	.22^b^

^a^N/A: not applicable.

^b^Wilcoxon signed rank test to test no difference in the distribution between the preintervention test and postintervention test scores.

#### IPQ Assessment

**Figure 6:**
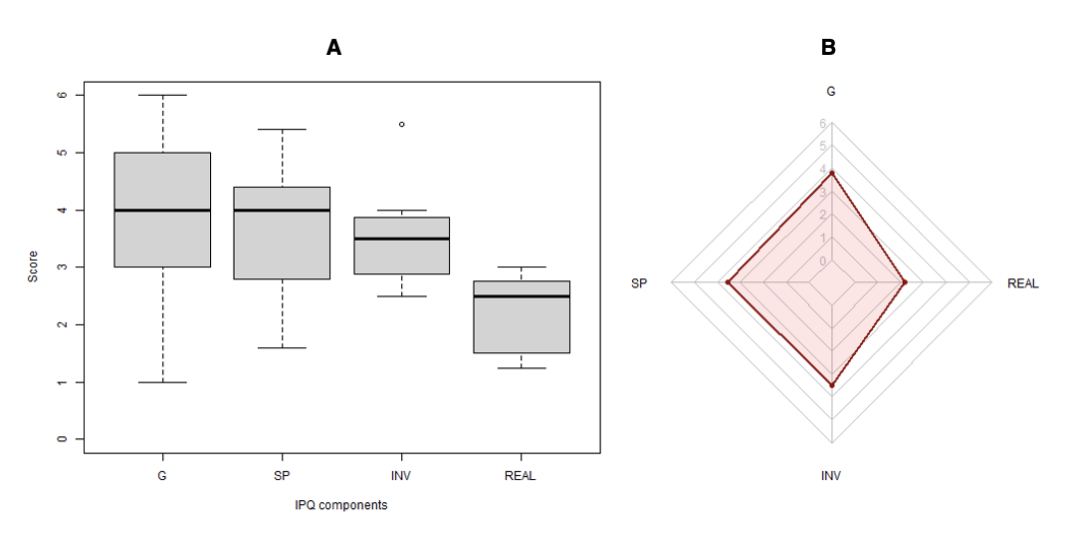
(A) Box plot showing median, quartiles, extreme data points, and outlier. (B) Radar chart of the overall mean of the 4 Igroup Presence Questionnaire (IPQ) components. It uses a radial axis, with the center representing a score of 0 and the outer outline representing a score of 6. G: general presence; INV: involvement; REAL: experienced realism; SP: spatial presence.

On the basis of the data collected from the 15 participants, the VR scenario achieved an above-average degree of overall presence, spatial presence, and involvement, with slightly below-average realism (Table 3 and Figure 6). Considering that the presence component is influenced by the other 3 components, it makes sense that it has a higher variance and SD, which suggests an opportunity to improve the immersion of the VR scenario. The lowest scoring component was realism, with the lowest variance and SD. These findings are corroborated by the qualitative feedback provided during the debrief session, where only 5 (33%) of the 15 participants commented that the environment felt realistic and that they felt immersed in the experience. By contrast, 1 (7%) of the 15 participants stated that they found the environment more immersive than simulation with real people. The participants’ feedback also highlighted other areas for future improvement, particularly regarding the realism component, such as having less restrictive dialogues, making the ICU environment more crowded, improving the voice-over acting features, and having the ICU equipment show patients’ physiological data (eg, heart rate monitor).

**Table 3:** Igroup Presence Questionnaire data statistics.

	Values, mean (SD)	Values, median (IQR)	Variance
General presence	3.80 (1.47)	4.0 (2.0)	2.17
Spatial presence	3.53 (1.16)	4.0 (1.6)	1.34
Involvement	3.48 (0.78)	3.5 (1.0)	0.60
Experienced realism	2.20 (0.67)	2.5 (1.3)	0.45

#### Mobile Data Analysis

The dropout rates for the study app were very high. Of the 15 participants, 8 (53%) did not perform any survey, whereas 7 (47%) completed at least 1 questionnaire. Instead of answering surveys periodically, only 4 (27%) of the 15 participants had >1 set of survey results. There were not sufficient mobile data to provide informative analysis. In the future, an improved participant engagement strategy is needed to help optimize mobile data collection.

#### Post Hoc Sample Size Calculation

As an exploratory analysis, we calculated post hoc sample sizes using 2-tailed paired *t* tests with a significance level of .05 based on the mean differences in the MIOS and PSS scores observed between the respective scores at prebrief and debrief. The common SDs for each score were estimated using the observed larger SD among the 2 scores. The computed correlations between the 2 scores were used in this calculation. The sample sizes required to achieve a power of 80% were 41 and 95 participants based on the observed results for the MIOS and the PSS, respectively (Multimedia Appendix 2).

### Qualitative Analysis

#### Content Analysis

Content analysis was performed for 14 (93%) of the 15 participants because technical issues compromised the speech recording of the 15th participant. Common references to real-life experiences were recognized in the content analysis, with the most frequent themes being the following: the virtual characters’ choices during the experiment were too restrictive (10/14, 71%), feelings of some guilt or shame (8/14, 57%), no feelings of failure or being punished (7/14, 50%), no guilt or shame (6/14, 43%), need of organizational support to deal with the morally challenging situation presented in the experiment (7/14, 50%), numbness (5/14, 36%), and the VR scenario was immersive, real, or engaging (5/14, 36%). Of the 14 participants, 1 (7%; participant 13) provided contradictory responses to feelings of guilt and shame, once saying that they did experience these feelings and once saying that they did not. Furthermore, 2 (14%) of the 14 participants considered the learning experience about MD and MI valuable and useful to their daily practice. A complete summary of the content analysis is provided in Multimedia Appendix 3.

Participants also recommended some specific areas of improvement in the VR scenario; for example, the following suggestions were made by 1 (7%) of the 14 participants: the patient’s vital signs were at a normal range although he was experiencing respiratory failure, the skin color should be consistent with that of the participant (all virtual characters were White), the scenario was unrealistic because other interventions apart from the ICU ventilator should have been portrayed, and photographs of the patient should have been added to better customize the character’s appearance. Finally, 2 (14%) of the 14 participants reported not being able to relate to religious mentions of God in the VR scenario.

#### User Experience

Although only 3 (20%) of the 15 participants reported prior experience with VR headsets (Multimedia Appendix 4), there were no dropouts during the VR scenario (Figure 5). As we had expected that new VR users could potentially experience nausea or disorientation, participants were reminded multiple times during the prebrief that they could pause or stop the session at any moment. Having said that, of the 15 participants, 14 (93%) did not report any side effects; only 1 (7%) participant reported claustrophobia and slight anxiety at first, but these feelings quickly subsided, and the participant was able to complete the VR scenario without any further side effects or complaints. Finally, all participants agreed that the VR platform and scenario were easy to navigate (Multimedia Appendix 4). Regarding the debrief feasibility questionnaire, of the 15 participants, 6 (40%) agreed that they learned about MD and interventions, and 11 (73%) agreed that the knowledge about MD and interventions will help them perform better in real-life events (Multimedia Appendix 5). Although only 8 (53%) of the 15 participants agreed that the VR simulation managed to make them experience the same emotions as they would in a real-life event (Multimedia Appendix 5), during the qualitative debrief, common emotions cited included some guilt, shame, betrayal, and isolation, which are consistent with MD.

## Discussion

### Principal Findings

In this work, we developed a fully immersive VR scenario to emulate a real experience of a morally distressing situation by HCWs in a simulated ICU setting during the COVID-19 pandemic and assess its acute effects on physiological and psychological parameters as well as longer-term effects on MD. This was followed by an educational video on MD and MI and appropriate mitigation strategies for MD and finally a repetition of the VR scenario in a pretest-posttest design. Because of COVID-19 constraints that resulted in health care settings often being described as a *war zone* [42], HCWs have been particularly exposed to PMIEs in their work environment during this pandemic [4,7]. However, despite the attention it has gained over the last decade, the concept of MI remains poorly understood. VR is a promising strategy to investigate MI owing to its ability to provide highly controlled virtual environments, personalized and tailored experiences, and full control and monitoring of the participants by the research team. The VR scenario created by the research team involved a complex ethical problem that became unfortunately frequent owing to the strain of the pandemic: prioritizing which patients would receive vital support in the face of the shortage of essential equipment such as ventilators [6]. This situation may be considered morally distressing because participants may witness the transgression of some of their core moral values [12], but it is not considered severe enough to induce MI. To achieve our goals, we performed a thorough quantitative and qualitative analysis of the acceptability, easiness of use, tolerability, and utility of the VR technology using a head-mounted display. To the best of our knowledge, this study is the first to examine the feasibility of using an immersive VR scenario to investigate the psychobiological impacts of a moral stressor on HCWs, as well as to use physiological parameters to predict the severity of stress and symptoms of MD and MI.

The feasibility analysis showed high acceptability of the VR scenario among participants, with no dropouts occurring during the study. Although only one-fifth of the participants (3/15, 20%) had previously used VR, all participants reported that the VR technology was easy to use. Moreover, the tolerability was also high because only 1 (7%) of the 15 participants reported mild transient side effects (claustrophobia); no participants reported nausea, whereas other specific side effects (eg, headache and dizziness) were neither reported by participants nor inquired on by the research team. This finding aligns with the literature showing that the incidence rate of VR-induced side effects is low and ranges between 0.5% and 8% [43], with the most common side effects being nausea, eye strain, and dizziness [43]. Specifically, nausea is reported to have an incidence rate of 5.2% [44], whereas vomiting is considered a rare event with an incidence rate of approximately 2% [45]. These symptoms are defined as cybersickness, a form of motion sickness that may be experienced during immersive VR experiences [44]. In this study, we hypothesize that the lack of nausea and other symptoms of cybersickness may have been due to limited head motion during the VR scenario and to the relatively reduced duration (mean 26.3, SD 2.7, min) of the experiment [46].

Regarding the technical quality of our VR scenario, the IPQ results revealed that the scenario achieved a high degree of general presence and spatial presence, above-average involvement, and slightly below-average realism. Therefore, most of the participants felt immersed and involved in the virtual environment but reported that the experiment was not realistic enough (10/15, 67%). This lack of realism was corroborated by the content analysis, where only approximately one-third of the participants (5/14, 36%) felt that the scenario was immersive, real, or engaging. To improve the experience of realism in virtual hospital environments, future studies could address the limitations pointed out by participants in the qualitative debrief session, such as more realistic ICU settings with equipment displaying patients’ vital parameters and having ethnically diverse virtual characters to be more representative virtual avatars of participants.

Content analysis of the debriefing revealed that feelings of guilt, shame, betrayal, isolation, and failure were commonly reported; these are impairing moral emotions consistent with MD [7,17,47] and might suggest a violation of moral beliefs. This finding suggests that the VR scenario could acutely induce real experiences of mild MD. Interestingly, numbness was mentioned by approximately a third of the participants (5/14, 36%). This feeling could be considered as a consequence of not having real power in relation to a real-world experience; it may also represent an emotional consequence of being exposed to a PMIE [12,18]. We assume that numbness could be related to potential signs of the erosion of moral agency, not in relation to our intervention but to previous real-world experiences of prolonged and repeated stressors and moral stressors. The content analysis revealed that most of the participants (8/14, 57%) reported guilt and shame, which are feelings consistently related to the experience of MD [7,17]. This finding suggests that the moral stressor experienced during the VR scenario could successfully induce some degree of MD. In addition, half of the participants (7/14, 50%) expressed the need for organizational support, an aspect frequently related to MD. Participants suggested that there could be a greater emphasis on organizational dimensions in future simulations, given the expressed need and the alignment with past research on MD [48]. The findings from the content analysis supported our hypothesis that a VR scenario can be successfully used to elicit and discuss real-life experiences and emotions related to MD.

In contrast to the qualitative results, the quantitative analysis did not show significant changes in the MIOS scores between before and after the experiment. The PSS scores showed the same trend and were not significantly different from baseline, which contradicts our hypothesis that the VR scenario would significantly increase stress levels. Both the MIOS and the PSS focus on symptoms developed over the last month. Although participants were instructed to rate their symptoms at that specific moment, these scales might not have enough sensitivity to capture acute changes in stress and MD symptoms. Alternatively, the changes in MD symptoms may have not been severe enough to induce significant changes in the MIOS scores acutely. Combining our findings from the qualitative and quantitative analyses, we assumed that some degree of MD was experienced by most participants, but we believe that these symptoms were not severe enough to induce MI. This is an important ethical aspect because the VR scenario was designed by specialists in MD and MI to minimize the risk of inducing significant MD in participants.

As MI may develop in the long term, we additionally attempted to use a mobile app to monitor participants for stress and MD and offer psychological support during an 8-week follow-up. Unfortunately, a longitudinal analysis of MD during the follow-up was not possible owing to very low app compliance. It is possible that participants might have developed additional symptoms of MD during follow-up that otherwise could not be captured by our analysis. However, we believe that this is unlikely because no participants requested the psychological support offered in the study. Alternatively, the brief version of the MIOS might not have been sensitive enough to detect slight but important changes in MD that would otherwise be detected by its complete version or by another MD scale. Having said that, this study is a feasibility study with a small sample size, and such an implication is beyond the scope of this work. Finally, the MIOS is still under development; hence, future studies are needed to assess the validity of the MIOS and its brief version.

Mobile app retention proved to be challenging because more than half of the participants (8/15, 53%) did not use the study app, and less than one-third (4/15, 27%) completed at least 1 set of surveys. Our app engagement strategy was based solely on in-app automated reminders and was insufficient to promote participant retention. This finding is supported by recent literature that recommends a combination of different engagement strategies to optimize app use [49,50]. In addition, another possible explanation for the low compliance is that a user-centered design process was not adopted during app development; therefore, the study app may not be particularly targeted to HCWs as the end users [51,52]. Nevertheless, our results are in line with previous research that demonstrates that retention is frequently a great challenge in mobile health studies in both clinical and nonclinical samples [50,53].

Post hoc sample size calculations indicate that a 3-fold and 6-fold sample size is required to reach a power of 80% for the MIOS and the PSS, respectively. With a sample of only 15 participants, our results were underpowered, which may at least in theory explain the nonsignificance of our quantitative findings and the discrepancy between the qualitative and quantitative results. This study was developed during a critical period of the COVID-19 pandemic, with recruitment occurring between May 2021 and August 2021, when contact restrictions were very strict. As the VR intervention required in-person data collection, recruitment proved to be very challenging. Nevertheless, our sample size of 15 participants is appropriate for a preliminary analysis, considering previous VR studies published in PTSD and other mental health disorders [54-57]. Our post hoc sample size calculations may be useful to guide the design of future adequately powered studies using VR in the context of MD and MI.

### Limitations

This study has several limitations that must be considered. First, it is a pilot feasibility study with a single arm and a small sample size; thus, the results should be interpreted with due caution. Additional studies with a controlled design are necessary to assess the safety and effectiveness of VR interventions in the assessment of MD and MI. Second, stratification analysis by demographic variables was not possible owing to the reduced sample size; therefore, we were unable to compare symptoms of MI among different subpopulations (eg, nurses and physicians). In addition, our experiments were performed on a purposive sample of only HCWs, thus limiting the generalizability of our findings to other populations. Third, the debriefing methodology used may have also provided a different lens than a traditional qualitative interview or focus group. Fourth, the MIOS and the PSS were used outside of their time frame scope; additional studies should include assessments that focus on acute symptoms of stress and MD. Fifth, a standardized cybersickness scale to assess the side effects within the VR scenario, such as the Virtual Reality Sickness Questionnaire [58], was not used and might have caused underreporting of side effects in this study. Sixth and last, the low app engagement found during the 8-week follow-up hindered an analysis of any potential long-term consequences of the experiment related to MD. Considering that the symptoms of MI may have a late onset, this represents an important limitation to our findings.

### Conclusions

The COVID-19 pandemic has challenged the mental health of HCWs, with increased rates of distress, anxiety, and depression being reported. During patient care, ethically difficult situations became common and put frontline HCWs at risk of MD and MI. VR-based interventions are a promising method to address these limitations because they allow for the possibility of developing experiments in safe, personalized, and highly controlled environments. This pilot study investigated the feasibility of using a VR scenario to simulate the experience of a mild morally challenging event for HCWs during the COVID-19 pandemic and to examine participants’ physiological reactions to making morally difficult decisions in a virtual environment. Our results suggest the feasibility of using a VR scenario to simulate real experiences of morally stressful events and elicit genuine responses associated with MD with high acceptability and tolerability. In addition, our VR-based intervention demonstrated utility as a pedagogical tool for teaching possible ways to prevent and mitigate MD. Future studies should be conducted to further validate our findings in a larger sample.

## Appendix

Multimedia Appendix 1 Postintervention debrief interview guide.

Multimedia Appendix 2 Post hoc sample size calculations.

Multimedia Appendix 3 Individual summary of the most common themes in the content analysis of data of 14 participants.

Multimedia Appendix 4 Results of the virtual reality scenario feasibility questions.

Multimedia Appendix 5 Scores from the debrief feasibility questionnaire.

## Abbreviations

ECG: electrocardiography
HCW: health care worker
ICU: intensive care unit
IPQ: Igroup Presence Questionnaire
MD: moral distress
MI: moral injury
MIOS: Moral Injury Outcome Scale
PEARLS: Promoting Excellence and Reflective Learning in Simulation
PMIE: potentially morally injurious event
PSS: Perceived Stress Scale
PTSD: posttraumatic stress disorder
VR: virtual reality

## Data Availability

The data sets generated and analyzed during this study are available from the corresponding author upon reasonable request.
