# Supplementary material for: Digital Interventions for Stress Among Frontline Health Care Workers: Results From a Pilot Feasibility Cohort Trial
Source: JMIR Serious Games. 2024 Jan 9;12:e42813. doi: 10.2196/42813 (PMC10783335; doi:10.2196/42813)
Supplement: Multimedia Appendix 1 [file games_v12i1e42813_app1.docx]

Multimedia Appendix 1: Postintervention debrief interview guide.

Debrief

- Thank you for participating in this VR simulation experiment

- Your VR sessions were recorded as videos and we will replay parts of them so that you will have the opportunity to reflect on your actions and share your thinking processes and emotions

- We hope that you can also provide constructive feedback for our experiment

- How was your overall experience? Can you comment specifically on the tech, decision

making and your emotions?

*Researcher proceeds to show the VR simulation in a 2D platform. Users will participate in a

semi-structured interview where users will answer the scenario checkpoints and questions in an

open-ended manner. Open-ended questions are dependent on the scenario and dialogue script.*

Scenario Debriefing using PEARLS - Guide for debrief:

1) Setting the Scene

● The goal of this VR simulation was to elicit moral distress and injury by creating a high stress scenario that occurred that resulted in the death of a patient due to COVID-19. The simulation was also created to explore the effectiveness of a virtual intervention to diminish moral distress and injury.

2) Reactions

● How do you feel after participating in the intervention?

○ (May need to probe for guilt, shame, anger, anxiety, helplessness, and relief.)

● Describe any of your moral beliefs that were violated.

● Describe things that happened that prevent you from acting the way you thought

you should.

3/4) Description & Analysis (to develop shared understanding of the scenario)

● You experienced a scenario that involved a violation of your moral beliefs and values because you were asked to withdraw treatment prematurely and without the family’s consent.

● You also experienced in the beginning of the scenario a lack of support/even the betrayal of your colleagues.

5) Application and Summary

● What aspects of this VR simulation experience will you find useful/not useful for your professional work? What are the takeaways? (participant/learner centred)

● We could also summarize key learning points here. (researcher centred)

- Now that we have finished the video review and the debrief, we will continue with feedback about our experiment.

*Proceeds with open-ended questions and 3 survey questions related to the experiment*

Open-ended feedback

- What suggestions or feedback would you give to improve the scenarios? Please comment on what can be improved, what can be more realistic and any deviation from real-life applications.

- Could you share something that you have learned about moral injury today? How might this apply to your clinical practice?

Survey questions

- Please rate how well you agree with the following statements from 1 to 5 (1 is strongly disagree, 5 is strongly agree)

*Researcher notes the participant’s answers*

1. I have learned a lot of new material about moral distress and interventions

| **Strongly disagree** | **Disagree** | **Neutral** | **Agree** | **Strongly agree** |
| --- | --- | --- | --- | --- |
| 1 | 2 | 3 | 4 | 5 |

1. Knowledge of moral distress and intervention help you perform better in real-life events

| **Strongly disagree** | **Disagree** | **Neutral** | **Agree** | **Strongly agree** |
| --- | --- | --- | --- | --- |
| 1 | 2 | 3 | 4 | 5 |

1. The VR simulation managed to make me experience the same emotions as I would in a real-life event

| **Strongly disagree** | **Disagree** | **Neutral** | **Agree** | **Strongly agree** |
| --- | --- | --- | --- | --- |
| 1 | 2 | 3 | 4 | 5 |
