# Supplementary material for: Digital Interventions for Stress Among Frontline Health Care Workers: Results From a Pilot Feasibility Cohort Trial
Source: JMIR Serious Games. 2024 Jan 9;12:e42813. doi: 10.2196/42813 (PMC10783335; doi:10.2196/42813)
Supplement: Multimedia Appendix 2 [file games_v12i1e42813_app2.docx]

Multimedia Appendix 2. Post hoc sample size calculations.

**Notes:**

Sample sizes were calculated for two-sided paired t-test at a significance level of .05 (α=.05) to test the null hypothesis, H_0_: pre-score = post-score i.e., no difference in the scores before and after the intervention.

**1. Sample size calculation for MIOS**

* Parameters were estimated from our pilot study as mean difference = 3.13; SD of each score = 9.86; and correlation of pre- and post-scores = 0.75.

| Power | Total sample size |
| --- | --- |
| 80% | 41 |
| 90% | 55 |

**2. Sample size calculation for PSS**

* Parameters were estimated from our pilot study as mean difference = 1.80; SD of each score = 8.10; and correlation of pre- and post-scores = 0.71.

| Power | Total sample size |
| --- | --- |
| 80% | 95 |
| 90% | 126 |
