# Supplementary material for: Digital Interventions for Stress Among Frontline Health Care Workers: Results From a Pilot Feasibility Cohort Trial
Source: JMIR Serious Games. 2024 Jan 9;12:e42813. doi: 10.2196/42813 (PMC10783335; doi:10.2196/42813)
Supplement: Multimedia Appendix 3 [file games_v12i1e42813_app3.docx]

Multimedia Appendix 3. Individual summary of the most common themes in the content analysis of data of 14 participants^a^.

| **Participant** | **P1** | **P2** | **P3** | **P4** | **P5** | **P6** | **P7** | **P8** | **P9** | **P10** | **P11** | **P12** | **P13** | **P14** |
| --- | --- | --- | --- | --- | --- | --- | --- | --- | --- | --- | --- | --- | --- | --- |
| **Theme** |  |  |  |  |  |  |  |  |  |  |  |  |  |  |
| Choices too restrictive | X |  | X | X | X |  | X | X |  | X | X | X | X |  |
| Some guilt and shame |  | X | X |  | X | X | X |  |  |  | X |  | X^b^ | X |
| No sense of being punished or failure |  | X | X | X | X |  |  | X |  |  |  |  | X | X |
| Organizational support needed | X |  | X | X |  | X | X | X |  |  |  |  | X |  |
| No guilt or shame | X |  |  | X |  |  |  |  | X | X |  | X | X^b^ |  |
| VR scenario immersive/real/ engaging | X |  |  |  | X |  | X |  |  |  |  |  | X | X |
| Numbness or lack of attention to emotions | X | X | X |  |  |  | X |  |  |  |  |  | X |  |
| Holds organization as the responsible party and lack of trust in organization |  |  |  |  | X | X |  |  |  |  |  |  | X |  |
| Learning was valuable |  | X |  |  | X |  |  |  |  |  | X |  |  |  |
| Felt betrayed |  |  |  |  |  |  |  |  |  | X |  |  |  | X |
| Did the best under the circumstances |  |  |  |  |  |  |  |  |  |  |  | X | X |  |
| Not really responsible because of provincial guideline |  |  |  |  |  |  |  |  |  |  | X |  |  |  |
| Some distress and disappointment |  |  |  |  |  |  |  | X |  |  |  |  |  |  |

^a^An X indicates that the participant has mentioned the corresponding theme during the debrief. Themes are shown from the most to the least frequent.

^b^Participant 13 provided contradictory responses.
