# Supplementary material for: Digital Interventions for Stress Among Frontline Health Care Workers: Results From a Pilot Feasibility Cohort Trial
Source: JMIR Serious Games. 2024 Jan 9;12:e42813. doi: 10.2196/42813 (PMC10783335; doi:10.2196/42813)
Supplement: Multimedia Appendix 4 [file games_v12i1e42813_app4.docx]

Multimedia Appendix 4. Results of the virtual reality scenario feasibility questions.

| **Participant ID** | **VR used before?** | **Nausea?** | **Ease of use?** |
| --- | --- | --- | --- |
| P1 | No | No | Yes |
| P2 | No | No | Yes |
| P3 | Yes | No | Yes |
| P4 | No | No | Yes |
| P5 | No | No | Yes |
| P6 | Yes | No | Yes |
| P7 | No | No | Yes |
| P8 | No | No | Yes |
| P9 | Yes | No | Yes |
| P10 | No | No | Yes |
| P11 | No | No | Yes |
| P12 | No | No | Yes |
| P13 | No | No | Yes |
| P14 | No | No | Yes |
| P15 | No | No | Yes |
