# Supplementary material for: Digital Interventions for Stress Among Frontline Health Care Workers: Results From a Pilot Feasibility Cohort Trial
Source: JMIR Serious Games. 2024 Jan 9;12:e42813. doi: 10.2196/42813 (PMC10783335; doi:10.2196/42813)
Supplement: Multimedia Appendix 5 [file games_v12i1e42813_app5.docx]

Multimedia Appendix 5. Scores from the debrief feasibility questionnaire.

| **Participant ID** | **Q1** | **Q2** | **Q3** |
| --- | --- | --- | --- |
| P1 | 4 | 4 | 4 |
| P2 | 4 | 4 | 4 |
| P3 | 3 | 4 | 3 |
| P4 | 3 | 5 | 3 |
| P5 | 4 | 4 | 4 |
| P6 | 4 | 4 | 3 |
| P7 | 4 | 4 | 3 |
| P8 | 3 | 4 | 4 |
| P9 | 3 | 4 | 4 |
| P10 | 3 | 4 | 4 |
| P11 | 3 | 4 | 4 |
| P12 | 2 | 2 | 1 |
| P13 | 4 | 5 | 2 |
| P14 | 3 | 4 | 4 |
| P15 | 2 | 2 | 1 |

**Question legend:**

Q1. I have learned a lot of new material about moral distress and interventions.

Q2. Knowledge of moral distress and intervention help you perform better in real-life events.

Q3. The VR simulation managed to make me experience the same emotions as I would in a real-life event.

**Scale legend:**

1. Strongly disagree.
2. Disagree.
3. Neutral.
4. Agree.
5. Strongly agree.
